# Advanced Imaging in Dental Research: From Gene Mapping to AI Global Data

**DOI:** 10.1177/00220345241293040

**Published:** 2024-10-27

**Authors:** D.T. Graves, S.E. Uribe

**Affiliations:** 1 Department of Periodontics, School of Dental Medicine, University of Pennsylvania, Philadelphia, PA, USA; 2 Department of Conservative Dentistry and Oral Health, Riga Stradins University, Riga, Latvia; 3 Baltic Biomaterials Centre of Excellence, Headquarters at Riga Technical University & RSU Institute of Stomatology, Riga, Latvia; 4 Department of Conservative Dentistry and Periodontology, LMU Hospital, LMU, Munich, Germany

**Keywords:** artificial intelligence, biofilm(s), biomaterial(s), cell signaling, computer vision/convolutional neural networks, digital imaging/radiology

## Abstract

Advances in imaging technologies combined with artificial intelligence (AI) are transforming dental, oral, and craniofacial research. This editorial highlights breakthroughs ranging from gene expression mapping to visualizing the availability of global AI data, providing new insights into biological complexity and clinical applications.

Advances in imaging techniques have revolutionized dental, oral, and craniofacial (DOC) research and clinical practice. From the early discovery of X-rays by Wilhelm Roentgen, which earned the first Nobel Prize, to the development of sophisticated tools such as computed tomography and magnetic resonance imaging, these innovations have transformed our ability to visualize and understand complex biological structures and pathologies in unprecedented detail. Today, these imaging techniques allow us to examine the human body in exquisite detail with only a portion visualized, leaving significant data for further exploration and insights, such as through radiomics (Gillies et al. 2016). The continued advancement of imaging technologies—combined with artificial intelligence (AI)—is opening up new ways to analyze this vast amount of data to uncover patterns undetectable by human experts (Schwendicke and Krois 2022a). The future of cross-annotation, combining clinical images with genomic and multimodal data, will help define an era of dentistry that is more preventive, personalized, participatory, and predictive (Schwendicke and Krois 2022b).

Over the past 3 y, advances in spatial transcriptomics, high-throughput omics, and advanced imaging technologies have provided new insights into the cellular organization of tissues and biofilms at the molecular and cellular levels. This special issue highlights some of these advances as they provide new insights into oral biology. Spatial transcriptomics, as shown by Haller et al. (2024), integrate global gene expression data with histological analysis, enabling the precise localization of gene expression patterns within tissues. This has been particularly valuable for studying craniofacial development and disease pathogenesis. James et al. (2024) proposed that the development of new computational methods, particularly machine learning, has greatly expanded the potential of imaging and omics technologies to facilitate the analysis of massive datasets and accelerate the identification of cellular and molecular patterns. Chatzopoulou et al. (2024) highlighted the combination of noninvasive 3-dimensional (3D) imaging techniques with fluorescence microscopy to monitor dental pulp stem cell grafts, providing real-time assessment of tissue integration and vascular development. Woo et al. (2024) further demonstrated the use of ultrasonography to noninvasively assess gingival vascular changes during inflammation, with implications for monitoring periodontal disease progression. Piña et al. (2024) demonstrated that spatial transcriptome analysis offers high-resolution insights into gene function within specific tissue compartments, advancing developmental biology and gene regulation. Together, these technologies reshape our ability to investigate biological complexity, from developmental processes to clinical applications in regenerative medicine. Ramirez-Puebla et al. (2024) showed that advances in plastic embedding and sectioning significantly enhanced probe penetration and facilitated the visualization of 3D biofilm structures. Volumetric reconstructions provide novel insights into the spatial organization of the human oral microbiome, which provides a better understanding of bacterial-bacterial interactions. Zhang et al. (2024) extended this field by using terahertz scanning near-field optical microscopy to observe cariogenic microbial domains and extracellular polysaccharide matrix formation in unprecedented detail, providing novel insights into the interactions that drive caries progression. In an innovative application, Benz et al. (2024) used shortwave infrared transillumination (SWIR) to enhance the visualization of pulpal structures during dental procedures. Their findings showed that SWIR provides clearer images than conventional methods do, potentially improving the precision and safety of endodontic treatment. This technology could reduce the risks associated with endodontic treatments by providing more accurate visualization of internal tooth structures.

Integrating AI into imaging technologies is transforming the processing and interpretation of vast datasets generated by advanced imaging methods. AI enables the detection of patterns in complex data, often surpassing human capabilities in terms of speed and precision. In dental, oral, and craniofacial research, AI applications promise to enhance diagnostic accuracy and radically change clinical workflow and treatment pathways. By linking imaging data with genomics and clinical annotations, AI opens the door to more predictive, preventive, and personalized care. However, high-quality clinical images are required to begin cross-annotating these datasets effectively. One source of high-quality clinical images is intraoral scanners. Here, Eggmann and Blatz (2024) reviewed the latest advances in intraoral scanners, their interoperability, and their integration with deep neural networks to facilitate the workflow of key orthodontics, surgery, and rehabilitation applications.

In this regard, van Nistelrooij et al. (2024) introduced JawFracNet, a model comparable to oral and maxillofacial surgeons for detecting jaw fractures but with the added benefit of faster detection. Also, Liu et al. (2024) proposed Geo-Net, a self-supervised pretraining framework that significantly boosts 3D tooth segmentation accuracy using large-scale unlabeled data. Both studies adopt open science practices and share data or codes to promote reproducibility and further research. Data are key to developing AI solutions in health care; however, they still need to be improved (Uribe et al. 2022). Uribe et al. (2024) from the WHO/ITU/WIPO Global Initiative for AI in Health comprehensively reviewed publicly available dental image datasets to develop AI tools. Their analysis identified 16 datasets but highlighted significant limitations, including data scarcity, inconsistent metadata reporting, and limited geographic diversity. This study also raises important concerns regarding data bias, data shift, and the generalizability of AI models across diverse populations.

These studies demonstrate the growing role of advanced imaging technologies and AI in dental, oral, and craniofacial research. The next challenge, however, is how to translate these advances into clinical practice as quickly, safely, and equitably as possible to align with the G2030 goal of reducing the prevalence of oral diseases by 10% while improving population coverage to 75% (World Health Organization 2024). This endeavor will require continued collaboration, innovation, and alignment between the different actors to ensure cutting-edge technologies reach clinical settings and reduce health inequities, benefiting patients worldwide.

## Author Contributions

D.T. Graves, S.E. Uribe, contributed to conception, design, data acquisition, analysis, and interpretation, drafted and critically revised the manuscript. All authors gave final approval and agree to be accountable for all aspects of the work.

## References

[bibr1-00220345241293040] Benz L Heck K Hevisov D Kugelmann D Tseng PC Sreij Z Litzenburger F Waschke J Schwendicke F Kienle A , et al. 2024. Longitudinal monitoring of alveolar bone regeneration using 3D imaging techniques. J Dent Res. 103(13):00–00. 10.1177/00220345241262949 PMC11633072 39101558

[bibr2-00220345241293040] Chatzopoulou E Bousaidi N Guilbert T Rucher G Rose J Germain S Rouzet F Chaussain C Muller L Gorin C. 2024. Multiscale imaging to monitor functional SHED-supported engineered vessels. J Dent Res. 103(13):00–00. 10.1177/00220345241271122 PMC11653307 39290146

[bibr3-00220345241293040] Eggmann F Blatz MB . 2024. Recent advances in intraoral scanners. J Dent Res. 103(13):00–00. 10.1177/00220345241271937 PMC11633065 39382136

[bibr4-00220345241293040] Gillies RJ Kinahan PE Hricak H. 2016. Radiomics: images are more than pictures, they are data. Radiology. 278(2):563–577. 26579733 10.1148/radiol.2015151169 PMC4734157

[bibr5-00220345241293040] Haller J Abedi N Hafedi A Shehab O Wietecha M. 2024. Spatial transcriptomics unravel the tissue complexity of oral pathogenesis. J Dent Res. 103(13):00–00. 10.1177/00220345241271934 PMC11653320 39382116

[bibr6-00220345241293040] James E Justo Caetano A Sharpe P. 2024. Computational methods in oral and craniofacial imaging. J Dent Res. 103(13):00–00. 10.1177/00220345241265048 PMC11633063 39272216

[bibr7-00220345241293040] Liu Y Liu X Yang C Yang Y Chen H Yuan Y. 2024. Geo-net: geometry-guided pre-training for tooth point cloud segmentation. J Dent Res. 103(13):00–00. 10.1177/00220345241292566 PMC11653261 39548729

[bibr8-00220345241293040] Piña J Raju R Roth D Winchester E Padilla C Iben J Faucz F Cotney J D’Souza R. 2024. Artificial intelligence for periodontal disease detection using deep learning. J Dent Res. 103(13):00–00. 10.1177/00220345241256600 PMC11653329 38910391

[bibr9-00220345241293040] Ramirez-Puebla ST Mark Welch JL Borisy GG . 2024. Improved visualization of oral microbial consortia. J Dent Res. 103(13):00–00. 10.1177/00220345241251784 PMC11653304 38828615

[bibr10-00220345241293040] Schwendicke F Krois J. 2022a. Data dentistry: how data are changing clinical care and research. J Dent Res. 101(1):21–29. 34238040 10.1177/00220345211020265 PMC8721539

[bibr11-00220345241293040] Schwendicke F Krois J. 2022b. Precision dentistry—what it is, where it fails (yet), and how to get there. Clin Oral Investig. 26(4):3395–3403. 10.1007/s00784-022-04420-1 PMC8918420 35284954

[bibr12-00220345241293040] Uribe SE Issa J Sohrabniya F Denny A Kim N Dayo A Chaurasia A Sofi-Mahmudi A Büttner M Schwendicke F. 2024. Publicly available dental image datasets for artificial intelligence. J Dent Res. 103(13):00–00. 10.1177/00220345241272052 PMC11633071 39422586

[bibr13-00220345241293040] Uribe SE Sofi-Mahmudi A Raittio E Maldupa I Vilne B. 2022. Dental research data availability and quality according to the FAIR principles.J Dent Res. 101(11):1307–1313. 35656591 10.1177/00220345221101321 PMC9516597

[bibr14-00220345241293040] van Nistelrooij N Schitter S van Lierop P El Ghoul K König D Hanisch M Tel A Xi T Thiem D Smeets R , et al. 2024. Detecting mandible fractures in CBCT scans using three-stage neural network. J Dent Res. 103(13):00–00. 10.1177/00220345241256618 PMC11633064 38910411

[bibr15-00220345241293040] Woo J Kripfgans O Wang IC Samal A Rodriguez Betancourt A Fenno JC Chan HL . 2024. Ultrasonographic evaluation of gingival vascular change during inflammation. J Dent Res. 103(13):00–00. 10.1177/00220345241286807 PMC11653338 39582155

[bibr16-00220345241293040] World Health Organization. 2024. Global strategy and action plan on oral health 2023–2030; [accessed 2024 Sep 27]. https://www.who.int/publications/i/item/9789240090538.

[bibr17-00220345241293040] Zhang A Lei L Cheng L Yin H Zhang C Luo J Wu F Hu M Cheng R Hu T. 2024. Terahertz imaging detects oral cariogenic microbial domains characteristics. J Dent Res. 103(13):00–00. 10.1177/00220345241287733 PMC11653314 39569646

